# Successful treatment of feline infectious peritonitis-associated myocarditis in a cat

**DOI:** 10.1177/20551169251366442

**Published:** 2025-10-17

**Authors:** Ewelina Korzybska, Conor O’Halloran, Geoff Culshaw, Nina Milevoj, Ana Fernandez-Gallego, Maria Ines Oliveira

**Affiliations:** Hospital for Small Animals, Royal (Dick) School of Veterinary Studies, University of Edinburgh, Easter Bush Campus, Midlothian, UK

**Keywords:** Feline infectious peritonitis, myocarditis, alpha-1 acid glycoprotein, troponin, GS-441524

## Abstract

**Case summary:**

A 4-year-old, indoor-only male castrated domestic shorthair cat was referred after identification of cardiomegaly and pleural effusion by the primary veterinarian. Echocardiography revealed generalised left ventricular hypertrophy with left atrial enlargement, pleural effusion and a small amount of pericardial effusion. Therefore, congestive heart failure was suspected, and the patient was treated with furosemide (2 mg/kg PO q12h). Subsequent investigations included pleural fluid analysis, plasma cardiac troponin I and serum alpha-1 acid glycoprotein (AGP) measurements. Plasma cardiac troponin concentration raised suspicion of myocarditis at 1.31 ng/ml (reference interval [RI] <0.05), while pleural fluid analysis revealed it to be a highly proteinaceous (84.3 g/l) exudate with mixed neutrophilic and macrophagic inflammation. A quantitative RT-PCR for feline coronavirus performed on the same fluid was positive in conjunction with markedly elevated serum AGP levels (2709 µg/ml; RI <500); therefore, a diagnosis of effusive feline infectious peritonitis (FIP) was made. The patient was treated with GS-441524 (10 mg/kg PO q12h) for 12 weeks, which resulted in resolution of the clinical signs, normalisation of AGP and fully reversed cardiac remodelling.

**Relevance and novel information:**

This case report summarises an unusual case of FIP as the putative cause of myocarditis in a cat. Although acute myocarditis has been well described in people as a cardiovascular complication of systemic coronavirus disease (COVID-19), this is the first suspected ante-mortem diagnosis in a cat with FIP. Furthermore, once the FIP was successfully treated, the cardiac abnormalities entirely resolved. This case also highlights the importance of pleural fluid analysis in cats with effusion, even when heart failure is suspected as the cause.

## Case description

A 4-year-old castrated male domestic shorthair cat was referred to our cardiology service with suspected congestive heart failure (CHF), cardiomegaly and pleural effusion (PE). The patient had never travelled outside the UK and was up to date with core vaccinations and parasite prevention.

The cat had been presented to his primary veterinarian 1 week earlier with hyporexia, 1 kg (25%) weight loss and acute-onset dyspnoea. A thoracic point-of-care ultrasound scan revealed cardiomegaly and PE. Thoracocentesis yielded 40 ml of ‘straw-coloured’ fluid that had not been analysed further.

Haematological and serum biochemical analyses had revealed decreased haematocrit (24%; reference interval [RI] 25–45) and total thyroxine concentration (T4 10 nmol/l; RI 13.4–48). The serum albumin concentration was decreased at 21 g/ (RI 26–40) while the globulin concentration was increased at 88 g/l (RI 26–51), resulting in an albumin:globulin ratio of 0.23 (RI 0.53–1.36). The serum concentration of N-terminal prohormone of brain natriuretic peptide (NT-proBNP) was increased at 719 pmol/l (RI <100). Referral was requested after only mild improvement after 7 days of diuresis with furosemide (2 mg/kg PO q12h).

On assessment at the referral centre, the patient’s respiratory rate was 42 breaths/min with increased effort. The cat weighed 4.3 kg with a body condition score of 3/9 (normal 4–5/9). The cat’s heart rate was 180 beats/min with a regular rhythm, and there was a grade III/VI left parasternal systolic heart murmur. The rest of the physical examination was unremarkable.

On Doppler echocardiography, the heart rate was in the range of 200–230 bpm. There was a generalised increased left ventricular (LV) end-diastolic wall thickness in the range of 5.8–6.6 mm (RI <6)^1^ and left atrial (LA) enlargement (left atrium:aorta ratio [LA:Ao] 2.1; RI ⩽1.5; maximum LA diameter on long axis 20.2 mm; RI <16.5).^1^ Left atrial fractional shortening (LAFS) was reduced (21%; RI >25) as shown in Table 1.^2^ There was mild mitral insufficiency, and E and A waves were summated on transmitral flow, reaching 0.9 m/s (RI <1.2).^3^ Systolic anterior motion of the mitral valve was also detected. Left ventricular outflow tract velocities were within the normal range (1.9 m/s; RI <2.7)^4^ but with a scimitar shape, suggestive of dynamic obstruction. There was moderate PE and trivial pericardial effusion without tamponade. The furosemide dosage was initially increased to 2 mg/kg PO q8h because of a suspected lack of control of CHF.

Arterial blood pressure, measured using the oscillometric method (tail) in sternal recumbency, was within the RI (123 mmHg systolic, 67 mmHg diastolic and 81 mmHg mean).^5^

Serum biochemistry revealed hypokalaemia (2.8 mmol/l; RI 3.5–5.2) with a normal creatinine concentration (91 µmol/l; RI 80–140). Plasma cardiac troponinI (cTnI) concentration was significantly elevated at 1.31 ng/ml(RI <0.05).

Thoracocentesis was repeated for therapeutic and diagnostic purposes, and yielded 50 ml of clear, viscous yellow fluid. The specific gravity was 1.044 with total solids of 86 g/l and nucleated cell count of 5.9 ×10^9^/l. Cytological examination revealed neutrophilic and macrophagic inflammation of moderate cellularity, consistent with a proteinaceous transudate. No bacteria or neoplastic cells were identified. Further staining for infectious agents (eg, Ziehl–Neelsen stain) was not performed because the cat did not hunt and had never been fed a diet of raw meat.

Feline infectious peritonitis (FIP) was suspected. Serum alpha-1 acid glycoprotein (AGP) was markedly increased (2709 µg/ml; RI <500), strongly supporting the diagnosis.^6^ Immunocytochemistry for feline coronavirus (FCoV) was non-diagnostic due to strong background staining, probably related to the high protein content of the effusion. However, FCoV RT-PCR of the pleural fluid was positive. These results confirmed a diagnosis of FIP.

The patient was hospitalised for 1 week for FIP treatment and supportive care. An oral liquid BOVA-compounded formulation of GS-441524 was prescribed (10 mg/kg PO q12h) under the cascade and relevant drug use legislation for the jurisdiction of practice in the UK. Maropitant (1 mg/kg IV q24h) and transdermal mirtazapine (2 mg q24h) were administered because of hyporexia but were discontinued after 3 days when the patient’s appetite returned. Oral potassium supplementation (468 mg q12h) and furosemide (2 mg/kg q8h) were continued.

The patient’s demeanour and respiration also markedly improved. Briefly, follow-up echocardiography before discharge confirmed resolution of both pleural and pericardial effusion and slightly reduced LA size (LA in long axis 1.69 cm, LA:Ao 1.59). The LV wall thickness remained increased (values unchanged) but a reduction in heart rate unmasked an impaired relaxation pattern on transmitral flow (shown in Table 1).

**Table 1:** Selected echocardiographic measurements over time

Measurement	Echo 1	Echo 2 (7 days after initial echocardiogram)	Echo 3 (3 months after initial echocardiogram)	Echo 4 (4 months after initial echocardiogram)
LA major (cm)	2.02	1.69	1.59	1.48
LA:Ao	2	1.59	1.5	1.48
LAFS (%)	21	22	24	27
LV thickness* (mm)	6.6	6.6	5.2	4.8
TMF	Summated E and A waves (0.9 m/s)	Impaired relaxation pattern	Impaired relaxation pattern	Normal TMF pattern

* Mean of three measurements, including interventricular septum and free wall

Ao = aorta; LA = left atrium; LAFS = left atrial fractional shortening; LV = left ventricle; TMF = transmitral flow

The patient was discharged with a further 11 weeks of GS-441524 at 10 mg/kg PO q12h, and the furosemide was reduced to 2 mg/kg PO q12h. Potassium supplementation was discontinued upon hypokalaemia resolution. The owners were instructed to weigh the cat weekly and to adjust the GS-441524 dose to maintain a minimum of 10 mg/kg PO q12h. Rechecks were scheduled for 2, 6 and 12 weeks after the start of treatment. The first recheck comprised an update on the patient’s clinical signs and a clinical examination, which was unremark-able. The owner reported no increase in respiratory rate (24 breaths/min) or effort, and the cat had a good appetite. Point-of-care ultrasound revealed no effusions; there-fore, no treatment changes were made.

At the second recheck, the patient was continuously well with a normal sleeping respiratory rate (>30 breaths/min) and effort. The cat’s body weight had increased to 5.1 kg and the serum AGP concentration had normalised. The patient’s serum biochemistry revealed an increased alanine transaminase (173 IU/l; RI 30–60). Total serum solids remained increased at 90 g/l (RI 55–78) comprising an increased globulin concentration (60 g/l; RI 26–51), although the albumin:globulin ratio approached normality (0.5; RI 0.53–1.36). An increased urea (8.5 mmol/l; RI 3.5–8) was consistent with a pre-renal azotaemia related to diuresis.

On completion of the GS-441524 course, previously identified serum biochemistry changes had normalised. Serum AGP concentration remained within the RI. On Doppler echocardiography, increases in LV wall thickness were equivocal (5.2–5.6 mm; RI <6) while LA long axis (15.9 cm; RI <16.5) and LA:Ao (1.5) had normalised.^1^ LAFS had increased to 24% (previously 21%; RI >25),^2^ but transmitral flow remained consistent with an impaired relaxation pattern. Plasma cTnI levels were within the RI (0.01 ng/ml; RI <0.07). At this stage, a gradual discontinuation of furosemide treatment was implemented over 2 weeks, alongside owner monitoring of sleeping respiratory rate.

**Figure 1:**
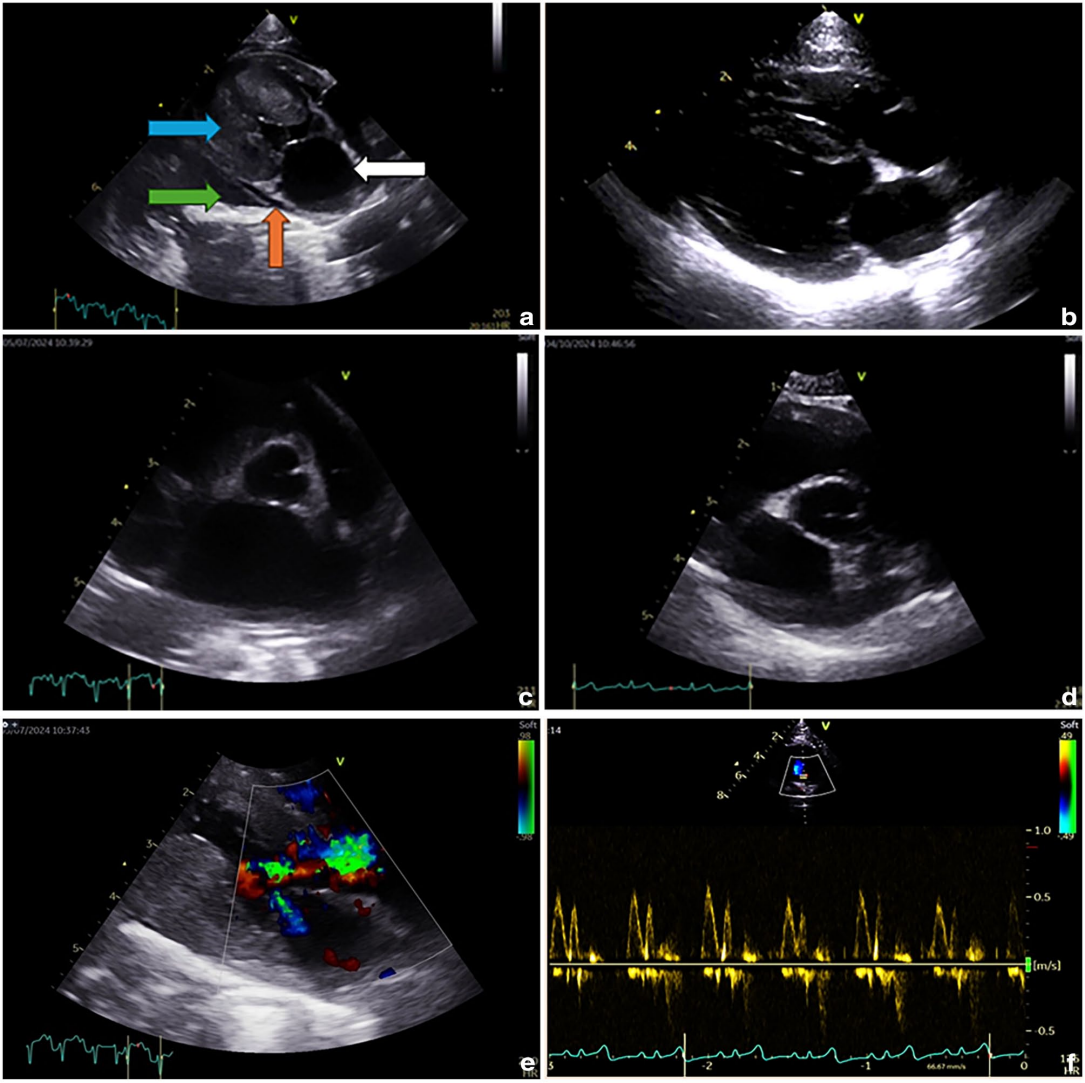
Selected echocardiographic images before and after treatment with GS-441524: (a) two-dimensional right parasternal long-axis view demonstrating generalised concentric hypertrophy (blue arrow), left atrial (LA) enlargement (white arrow), mild pleural effusion (green arrow) and a small amount of pericardial effusion (red arrow) at initial presentation; (b) end-diastolic two-dimensional right parasternal long-axis view showing resolution of previous changes, 4 months after initial presentation; (c) two-dimensional right parasternal short-axis view, at the level of the aortic valve (first frame of aortic valve closure), showing marked enlargement of the left atrium (left atrium:aorta ratio [LA:Ao] 2.1) at initial presentation; (d) two-dimensional right parasternal short-axis view, at the level of the aortic valve (first frame of aortic valve closure), showing normal LA size (LA:Ao 1.48); (e) two-dimensional right parasternal long-axis view with colour Doppler demonstrating turbulent flow in the left ventricular outflow tract and across the mitral valve at initial presentation; and (f) colour Doppler image obtained from the left apical four-chamber view showing normal diastolic function on transmitral flow.

At the final reassessment, 1 month later and 4 months after the first presentation, LA size, LV wall thickness and LAFS were within the RIs (LA long axis 14.8 mm; LA:Ao 1.48; LV wall thickness 4.8 mm; LAFS 27%), and transmitral flow was consistent with normal diastolic function (see Figure 1). The cat remained well at the time of writing, 3 months later.

## Discussion

This is the first recorded case of an ante-mortem diagnosis of myocarditis suspected to be associated with FIP. In humans, acute myocarditis is defined as a sudden inflammatory injury to the myocardium, affecting 4–14 out of 100,000 people each year globally, with a mortality rate of 1–7%.^7,8^ The most common causes of human myocarditis are viruses, such as influenza and coronavirus.^8^ Human coronavirus-associated myocarditis has become increasingly diagnosed in the last 5 years since the appearance of coronavirus disease 2019 (COVID-19).^9^ The pathophysiology of COVID-19-related myocarditis is thought to be a combination of direct viral injury alongside cardiac damage due to the exuberant host immune response.^8–11^ There is no evidence-based treatment specifically recommended for myocarditis; instead, patients are managed according to standard COVID-19 and heart failure treatment protocols.^12^

Similar to COVID-19 in humans, FCoV infection can lead to a severe systemic disease: FIP.^13,14^ Cats with FIP are usually young and are presented with various clin-ical signs, including effusions into body cavities (as in this patient) and/or pyogranuloma formation affecting almost any body system. In either situation, if left untreated, FIP is fatal.^6,15–17^ Since 2019, FIP has become treatable using antiviral medications such as GS-441524, with an 85% success rate.^17,18^ This cat was treated for 12 weeks, as has been previously described.^17,18^

Our patient was suspected of having myocarditis because of LV hypertrophy, increased cTnI and a positive response to treatment, which included reverse cardiac remodelling. The simultaneous diagnosis of FIP led to the strong clinical suspicion that this had contributed to the development of a form of myocarditis similar to transient myocardial thickening (TMT), which has been described previously.^19,20^ TMT also predominantly affects young cats, and is characterised by LA enlargement and dysfunction, increased cTnI and concurrent CHF, which subsequently resolves within weeks to months.^19,20^ These patients are often presented after a precipitating event (eg, anaesthesia) or as a secondary consequence of infection (eg, toxoplasmosis or sepsis).^19,20^ There was no history of a precipitating event preceding the acute presentation of our patient. We propose that FIP is a potential cause of myocarditis that resolves after treatment, bearing hallmarks similar to TMT.

Currently, there are three existing reports of cats where concurrent FIP and myocarditis were found on post-mortem examination,^21–23^ and one single case series reporting suspected SARS-CoV-2 B.1.1.7 variant myocarditis in six cats and one dog.^24^ In the individual case reports, myocarditis was confirmed using the reference standard of histopathology, and immunohistochemistry performed on the myocardium revealed FCoV-positive macrophages associated with pyogranulomatous lesions that confirmed FIP.^21,22^ In the case series describing SARS-CoV-2 B.1.1.7 variant myocarditis, diagnosis in all patients was based on a combination of clinical presentation and a positive result for SARS-CoV-2 PCR on rectal swab or the presence of anti-SARS-CoV-2 antibodies. Laboratory tests for diagnosing FIP were not performed in these animals. The patients were treated symptomat-ically with various outcomes.^24^ To the authors’ know-ledge, an ante-mortem diagnosis followed by successful treatment of FIP-related myocarditis has not been described before.

The patient was treated with furosemide for CHF, but it is possible that the effusion was exclusively due to FIP, which would have rendered diuretic therapy unnecessary. This theory is supported by the lack of initial response to diuresis. Based on marked LA enlargement and ongoing hospitalisation, the authors elected to continue diuresis until the LA size had reduced and the owners had been trained on how to monitor for respiratory decompensation, allowing for a gradual diuretic withdrawal.

In the human literature, LA enlargement is commonly reported in patients with COVID-19 myocarditis.^9,10^ LA enlargement may therefore represent a feature of FCoV-myocarditis phenotype rather than its haemodynamic consequence.

It remains possible that the initial increase in cTnI was secondary to the presence of pericardial effusion and consequent inflammation rather than primary myocarditis. It is also possible that myocarditis was caused by a separate coinfection; however, additional investigations were not performed because of the low incidence of the causative infections in the UK (eg, bartonellosis) and the absence of a history of hunting. Infiltrative neoplastic disease was initially considered to be a differential diagnosis; however, resolution of cardiac lymphoma without chemotherapy would be unlikely.^25,26^ Finally, cardiac muscle infiltration with secondary CHF has been reported in feline hypereosinophilic syndrome,^27^ but peripheral eosinophilia was never recorded in this case.

## Conclusions

This report describes the first successful treatment of a suspected case of myocarditis secondary to FIP. Importantly, the myocarditis and associated echocardiographic changes completely resolved with treatment of the FIP. The authors also wish to highlight the importance of performing fluid analysis in cats presented with any effusion, since appropriately treated FIP, as opposed to CHF, carries a better long-term prognosis.^17,18^
